# A Novel Risk Score Model for the Differential Diagnosis of Type 2 Diabetic Nephropathy: A Multicenter Study

**DOI:** 10.1155/2023/5514767

**Published:** 2023-12-21

**Authors:** Yuetong Zhao, Lin Liu, Li Zuo, Xianghai Zhou, Song Wang, Hongwei Gao, Feng Yu, Xiaomei Zhang, Mi Wang, Ling Chen, Rui Zhang, Fang Zhang, Shuhong Bi, Qiong Bai, Jiaxiang Ding, Qinghua Yang, Sixu Xin, Sanbao Chai, Min Chen, Junqing Zhang

**Affiliations:** ^1^Department of Endocrinology, Peking University First Hospital, Beijing, China; ^2^Department of Clinical Nutrition, Peking University First Hospital, Beijing, China; ^3^Department of Nephrology, Peking University People's Hospital, Beijing, China; ^4^Department of Endocrinology, Peking University People's Hospital, Beijing, China; ^5^Department of Nephrology, Peking University Third Hospital, Beijing, China; ^6^Department of Endocrinology, Peking University Third Hospital, Beijing, China; ^7^Department of Nephrology, Peking University International Hospital, Beijing, China; ^8^Department of Endocrinology, Peking University International Hospital, Beijing, China; ^9^Department of Nephrology, Peking University First Hospital, Beijing, China; ^10^Institute of Nephrology, Peking University, Beijing, China

## Abstract

**Introduction:**

DN is a common complication of diabetes. However, diabetes combined with renal injury may involve DN or NDKD, with different treatment schemes. The purpose of our study was to determine the independent risk factors of DN and establish a risk score model to help differentiate DN and NDKD, providing a reference for clinical treatment.

**Methods:**

A total of 678 T2D patients who had undergone renal biopsy in four affiliated hospitals of Peking University were consecutively enrolled. Patients were assigned to the DN group and NDKD group according to histopathological results. Seventy percent of patients from PKUFH were randomly assigned to the training group, and the remaining 30% were assigned to the internal validation group. Patients from the other three centers were assigned to the external validation group. We used univariate and multivariate logistic regression analyses to identify independent risk factors of DN in the training group and conducted multivariate logistic regression analysis with these independent risk factors in the training group to find regression coefficients “*β*” to establish a risk score model. Finally, we conducted internal and external validation of the model with ROC curves.

**Results:**

Diabetic retinopathy, diabetes duration ≥ 5 years, eGFR < 30 ml/min/1.73 m^2^, 24 h UTP ≥ 3 g, and no hematuria were independent risk factors (*P* < 0.05), and each factor scored 2, 1, 1, 1, and 1. We assigned the patients to a low-risk group (0-1 points), a medium-risk group (2-3 points), and a high-risk group (4-6 points), representing unlikely DN, possibly DN, and a high probability of DN, respectively. The AUCs were 0.860, 0.924, and 0.855 for the training, internal validation, and external validation groups, respectively.

**Conclusion:**

The risk score model could help differentiate DN and NDKD in a noninvasive manner, reduce the number of renal biopsies, and provide a reference for clinical treatment.

## 1. Introduction

Globally, approximately 536.6 million (10.5%) people had diabetes in 2021, and the number is expected to rise to 783.2 million (12.2%) by 2045 [1]. Diabetic nephropathy (DN) develops in approximately 40% of patients with type 2 diabetes (T2D) and is the leading cause of end-stage kidney disease (ESKD) [2]. The rate of ESKD remains unchanged despite a decline in other diabetes complications [3]. A better understanding of the characteristics of DN is thus warranted to potentially prevent the progression from DN to ESKD.

In clinical situations, patients with T2D accompanied by chronic kidney disease (CKD) are commonly seen. Apart from DN, nondiabetic kidney disease (NDKD) accounts for a large proportion of cases. A review identified 40 studies (5304 data) worldwide from 1977 to 2019 that examined global renal biopsy in type 2 diabetes patients, and the overall prevalence rates of DN, NDKD, and DN plus NDKD were reported to be 41.3%, 40.6%, and 18.1%, respectively [4]. NDKD (including mixed form) manifests a wide spectrum of pathological lesions across the world, including focal segmental glomerulosclerosis (FSGS), membranous nephropathy (MN), and immunoglobulin A nephropathy (IgAN) [5]. The management of DN has evolved from a simple glycemic management perspective to a multifactorial intensive treatment perspective that is different from various NDKD management approaches [6]. Therefore, it is important to distinguish DN from all CKDs. At present, differentiation between DN and NDKD mainly depends on the gold standard—ultrasound-guided renal biopsy. However, it is an invasive examination, and many patients are unable to undergo it because of abnormal coagulation function, kidney atrophy, isolated kidney, or severe cardiopulmonary insufficiency. Thus, a noninvasive differentiation method is needed.

In our study, by retrospectively analyzing the clinical data and histopathological results of T2D patients, we compared the differences between the clinical characteristics of DN and NDKD, analyzed the clinical characteristics and risk factors of DN, and then established a DN risk score model for T2D patients to help clinicians better differentiate DN and NDKD, providing a reference for clinical treatment.

## 2. Materials and Methods

### 2.1. Patients

**Figure 1:**
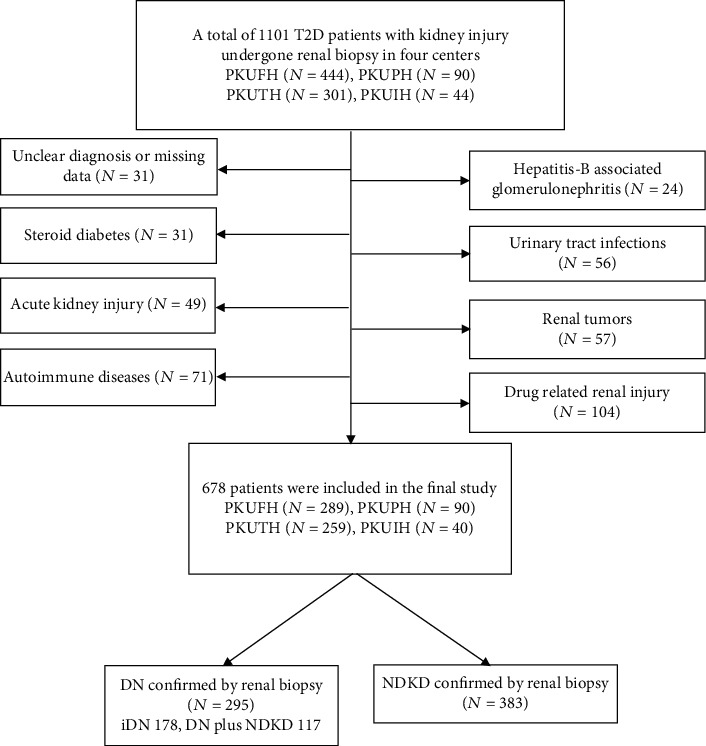
Flow chart of the patient enrollment process. PKUFH: Peking University First Hospital; PKUPH: Peking University People's Hospital; PKUTH: Peking University Third Hospital; PKUIH: Peking University International Hospital; T2D: type 2 diabetes; DN: diabetic nephropathy; iDN: isolated diabetic nephropathy; NDKD: nondiabetic kidney disease.

We consecutively recorded 1,101 patients with T2D who underwent percutaneous renal biopsy from January 1, 2018, to December 31, 2019, at Peking University First Hospital (PKUFH) (289 patients) and from January 1, 2012, to December 31, 2020, at Peking University People's Hospital (PKUPH) (90 patients), Peking University Third Hospital (PKUTH) (259 patients), and Peking University International Hospital (PKUIH) (40 patients). Patients with type 1 diabetes, steroid diabetes and other special types of diabetes, acute kidney injury, autoimmune diseases such as systemic lupus erythematosus, Sjogren's syndrome, amyloidosis, hepatitis B-associated glomerulonephritis, renal tumors, drug-related renal injury, urinary tract infections, and unclear diagnoses or missing data were excluded (Figure 1). Finally, 678 patients were enrolled in our study, including 295 DN patients and 383 NDKD patients based on histopathological results. Each center's ethics committee approved the study after ethical review.

### 2.2. Data Collection

We collected patients' demographics, medical history, laboratory results, and renal histopathological results through the medical record system. The data on age, sex, body mass index (BMI), diabetes duration, diabetic complications, other chronic disease histories—such as primary hypertension and dyslipidemia, hemoglobin concentration, urine occult blood, serum creatinine, estimated glomerular filtration rate (eGFR), triglycerides, total cholesterol, urinary microalbumin (MA), albumin creatinine ratio (ACR), 24-hour urinary total protein quantification (24 h UTP), light microscopy, direct immunofluorescence, electron microscopy, etc. are shown in Table 1.

### 2.3. Renal Histopathology

The biopsy specimens were independently reviewed and scored by two experienced renal pathologists. Light microscopic sections were stained with hematoxylin and eosin, periodic acid-Schiff, Masson's trichrome, and periodic acid methenamine silver stain. The diagnostic standard of DN was the Tervaert grading standard [7].

### 2.4. Subgroup

According to the histopathological results, patients were assigned to a DN group and an NDKD group. The DN group contained 178 isolated DN (iDN) patients and 117 DN plus NDKD patients (Figure 1). NDKD included MN and IgA nephropathy. In total, there were 383 patients.

### 2.5. Statistical Analyses

Descriptive statistics are reported as percentages for categorical data, averages (mean ± SD) for continuous, normally distributed data, and medians (interquartile range, IQR) for continuous, nonnormally distributed data. The respective between-group comparisons were performed using *t* tests, *χ*^2^ tests, and the Mann–Whitney *U* tests. Independent associations between clinical variables and the risk of diabetic nephropathy were studied with logistic regression analysis. All data were analyzed using IBM SPSS Statistics for Windows version 20.0.

Seventy percent of patients from PKUFH were randomly assigned to the training group (202 patients), and the remaining 30% were assigned to the internal validation group (87 patients). Patients from the other three centers were assigned to the external validation group (389 patients).

We used univariate and multivariate logistic regression analyses to identify independent risk factors for DN in the training group (Table 2).

Multivariate logistic regression analysis was then conducted with the above independent risk factors in the training group to find the regression coefficients “*β*” to establish a risk score model (Table 3). The values of different factors were determined according to the *β* coefficients, and the total risk score was judged by the cumulative values of each factor. The risk score model was internally and externally validated using the validation groups with ROC curve and area under the ROC curve (AUC). The goodness-of-fit (GOF) of the model was tested by the Hosmer–Lemeshow method. All tests were conducted bilaterally, and *P* < 0.05 indicated a statistically significant difference.

## 3. Results

### 3.1. General Data

There were 678 patients included in our final study, including 467 males and 211 females, with an average age of 54.1 ± 11.8 (range 15-81) years. The median duration of diabetes was 6 (range 0~33) years. The average diabetes onset age was 46.5 ± 11.7 (range 14~78) years. A total of 542 patients (80.1%) had hypertension, 396 patients (58.4%) had hyperlipidemia, 111 patients (16.4%) had coronary heart disease, and 99 patients (14.6%) had previous cerebral vascular disease. A total of 311 patients (45.9%) had a smoking history, 237 patients (35.0%) had a drinking history, and 226 patients (33.3%) had a family history of T2D. There were 167 patients (57.7%) whose eGFR was <60 ml/min/1.73 m^2^ and 81 patients (28.0%) whose eGFR was <30 ml/min/1.73 m^2^. A total of 198 patients (29.2%) had negative urine occult blood. A total of 639 patients (94.2%) had proteinuria in routine urine tests. A total of 106 patients (106/663, 16.0%) had a 24-hour UTP of less than 1 g, 160 patients (160/663, 24.1%) had a 24-hour UTP of ≥1 g and <3 g, and 397 patients (397/663, 59.9%) had a 24-hour UTP of ≥3 g.

There was no significant difference in each variable between the training group and the internal validation group, except for 24 h UTP (*P* = 0.027) (data not shown). Differences between the DN and NDKD groups in the training group were similar to those in the total sample population, except for uric acid and drinking history. Data from the DN and NDKD groups of the training group, internal validation group, and external validation group are shown in Table 1.

### 3.2. Risk Factors

We explored the optimal cutoff values for all continuous variables and transformed them into binary variables. The specific cutoff values are shown in Table 2.

In the training group, we conducted univariate logistic regression and found significant differences in diabetic retinopathy, diabetes duration, eGFR, urea nitrogen, sex, diabetes onset age, 24 h UTP, hematuria, uric acid, smoking history, and hypertension history between the DN and NDKD groups.

Then, through multivariate logistic regression analysis, we found that diabetic retinopathy, diabetes duration ≥ 5 years, eGFR < 30 ml/min/1.73 m^2^, 24 h UTP ≥ 3 g, and no hematuria were independent risk factors of DN (*P* < 0.05) (Table 2).

### 3.3. Risk Score Model

Multivariate logistic regression analysis was reconducted with the above 5 independent risk factors in the training group. According to the regression coefficient “*β*,” 1 point was assigned for every ~1.1 increase (Table 3). According to *β*, diabetic retinopathy counted as 2 points, diabetes duration ≥ 5 years counted as 1 point, eGFR < 30 ml/min/1.73 m^2^ counted as 1 point, 24 h UTP ≥ 3 g counted as 1 point and no hematuria also counted as 1 point (Table 3). The total risk score was between 0 and 6. The patients were classified into low-risk (0-1 point), medium-risk (2-3 points), and high-risk (4-6 points) groups, representing unlikely DN, possibly DN, and high probability of DN, respectively. In the training group, the DN rates were 15.1% (11/73) in the low-risk group, 54.2% (38/70) in the medium-risk group, and 91.5% (54/59) in the high-risk group (Table 4).

### 3.4. Model Validation

#### 3.4.1. Internal Validation

**Figure 2:**
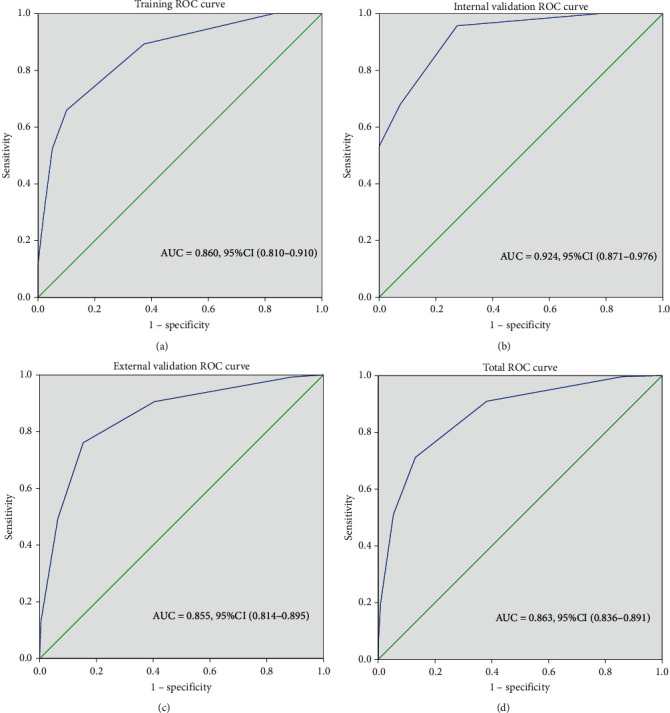
The ROC curves in each group. (a) ROC of the training group based on 202 patients, AUC = 0.860, 95% CI (0.810-0.910); (b) ROC of the internal validation group based on 87 patients, AUC = 0.924, 95% CI (0.871-0.976); (c) ROC of the external validation group based on 373 patients, AUC = 0.855, 95% CI (0.814-0.895); sixteen patients were included in the missing item because of incomplete data; (d) ROC of 662 patients, AUC = 0.863, 95% CI (0.836-0.891).

To verify the diagnostic efficiency of the model, the sensitivity and specificity of different cutoff points were calculated. In the internal validation group, when the cutoff point was 2, the sensitivity of the diagnosis of DN reached 0.957, the specificity was 0.725, and the Jordan index was 0.682. When the cutoff point was 4, the specificity of the diagnosis of DN reached 1. By drawing the ROC curve, AUC = 0.924 (*P* < 0.001, 95% CI 0.871-0.976) (Figure 2(b)). The GOF was good according to the Hosmer–Lemeshow test (*χ*^2^ = 1.863, *P* = 0.967). In the internal validation group, the DN rates were 6.5% (2/31) in the low-risk group, 64.5% (20/31) in the medium-risk group, and 100% (25/25) in the high-risk group (Table 4).

#### 3.4.2. External Validation

To further verify the diagnostic efficacy of the risk score model, we enrolled 389 patients from three other hospitals from 2012 to 2020 and applied the risk score model for external validation. In the external validation group, when the cutoff point was 2, the sensitivity of the diagnosis of DN reached 0.906, and the specificity was 0.596. When the cutoff point was 4, the specificity of the diagnosis of DN reached 0.936. By drawing the ROC curve, the AUC = 0.855 (*P* < 0.001, 95% CI 0.814-0.895) (Figure 2(c)). The Hosmer–Lemeshow test was further used to test the GOF of the model, which showed that the model had a good fitting effect (*χ*^2^ = 2.048, *P* = 0.727). In the external validation group, the DN rates were 8.5% (13/153) in the low-risk group, 41.6% (57/137) in the medium-risk group, and 81.9% (68/83) in the high-risk group (Table 4).

## 4. Discussion

According to a previous systematic review, not all T2D patients undergoing renal puncture had DN, 40.6% had NDKD, and 18.1% had DN plus NDKD [4]. Treatment plans for kidney diseases differ with different causes. In DN, the focus is more on the management of blood glucose and strengthening as well as the comprehensive management of related metabolic diseases, and the daily nutritional treatment of DN patients is also different from that of patients with other CKDs [6, 8]. In addition, DN progresses to ESKD faster than other kidney diseases [9]. Therefore, it is important to distinguish DN from all CKDs to guide clinical treatment.

In our study, hepatitis B-associated glomerulonephritis, drug-related renal injury, and other kidney diseases that were clinically easy to diagnose were excluded. However, there are still many NDKDs that are difficult to identify without renal biopsy, such as MN, IgA nephropathy, and ischemic nephropathy. In our study, NDKD accounted for 56.5% of the total population, and DN plus NDKD accounted for 17.3%, similar to previous reports.

As previous studies have shown [10, 11], the well-known predictors for the presence of DN (diabetic retinopathy, diabetes duration, and no hematuria) were also evident in our study. We found that the cutoff value of diabetes duration was 5 years, which is conducive to the earlier identification of DN compared with the “10 years” [12] or “15 years” [13] proposed in previous studies. This study found that eGFR < 30 ml/min/1.73 m^2^ was also an indicator, which may be related to the fact that the kidney function of DN patients deteriorates more rapidly than that of patients with other CKDs, thus reflecting a more rapid development and accounting for more cases of ESKD [12, 14, 15]. Nephrotic syndrome is a relatively common clinical manifestation of DN [16, 17], mainly manifested as massive proteinuria, which is consistent with the 24 h UTP ≥ 3 g found in our study, but massive proteinuria alone cannot indicate DN and may also signify membranous nephropathy, etc. [16], so the combination of the above five indicators is conducive to more accurate identification of DN. It has a higher accuracy than diabetic retinopathy or diabetes duration alone [18, 19].

In recent years, there have been intermittent studies on risk score models for DN diagnosis both domestically and internationally. Liu et al. [20] examined 200 patients with T2D who underwent renal biopsy and constructed a new diagnostic model as follows: PDN = exp (0.846 + 0.022 Dm + 0.033Bp + 2.050 Gh − 2.664 Hu − 0.078 Hb + 2.942Dr)/[1 + exp (0.846 + 0.022 Dm + 0.033 Bp + 2.050 Gh − 2.664 Hu − 0.078 Hb + 2.942 Dr)]. Validation tests determined that the accuracy of the new model was 90.9%. Zhou et al. [21] screened 110 diabetic patients with overt proteinuria but no severe renal failure for renal biopsy, and the diagnostic model was constructed as follows: PDN = exp (−13.5922 + 0.0371Dm + 0.0395Bp + 0.3224Gh–4.4552Hu + 2.9613Dr)/[1 + exp (−13.5922 + 0.0371Dm + 0.0395Bp + 0.3224Gh–4.4552Hu + 2.9613Dr)]. Zhang et al. [22] screened 1,030 patients with T2D who had undergone renal biopsy and selected ten variables to build the model using two machine learning methods. The models constructed by Liu and Zhou have a common problem, which is that the PDN formula is complex and not convenient for clinical application. It needs to be converted into a computer program to reflect its value. The model constructed by Zhang et al. contains ten variables and requires complex computer programs.

In our model, there were five variables categorized as 2 diabetes-related (diabetes duration and diabetic retinopathy) and 3 kidney-related (eGFR, 24 h UTP, and hematuria) items; these were all common indicators in clinical practice, even in most grassroots medical institutions. Our model score can be quickly calculated and ranked using simple manual methods. The model was verified internally and externally (spatially and temporally).

In our model, patients in the low-risk group had a 6.5% to 15.1% probability of DN, patients in the medium-risk group had a 41.6% to 64.5% probability of DN, and patients in the high-risk group had an 81.9% to 100% probability of DN. For high-risk group patients, it may be possible to reduce the number of renal biopsies.

All enrolled patients in our study were managed according to the latest guidelines during hospitalization. However, our study is a retrospective study with a wide time span. Since 2012, diabetes treatment drugs have also been updated. In recent years, with the application of kidney protective drugs such as sodium-glucose cotransporter-2 inhibitors (SGLT-2i) and mineralocorticoid receptor antagonists (MRAs) in clinical practice in China, the cutoff point values of DN duration in our model may change. For the same reason, although the main indications for renal biopsy in T2D patients involved in our study were rapid onset or progression of albuminuria or sudden onset of nephrotic syndrome, rapid eGFR decline, glomerular hematuria, active urine sediment, suspicion of other systemic diseases, and patients with type 1 diabetes, short diabetes duration and absence of retinopathy, etc., these indicators may vary slightly with the update of guidelines and individual complex situations that may arise in clinical practice [11, 23, 24]. These issues also exist in previous research models by Liu et al. [20–22]. Meanwhile, since DN patients with microalbuminuria do not undergo routine renal biopsy, this study found that, compared to our clinical experience, the DN group exhibited higher 24-hour UTP and poorer renal function. These factors cannot be avoided but may lead to selection bias when enrolling patients.

In addition, Pafundi et al. [25] showed that urine albumin excretion is associated with the highest cardiorenal risk. Therefore, early identification and intervention for DN with MA are more important. Reviewing the renal histopathological characteristics of T2D patients with MA alone and establishing a model may make the model more meaningful for the early diagnosis of DN and facilitate clinical treatment.

## 5. Conclusions

We found that diabetic retinopathy, diabetes duration ≥ 5 years, eGFR < 30 ml/min/1.73 m^2^, 24 h UTP ≥ 3 g, and no hematuria were independent risk factors for DN. Our study constructed a simple risk score model to identify DN patients from T2D patients with any renal injury. The risk score model was convenient to calculate and memorize and can help differentiate DN and NDKD in a noninvasive manner, reduce the number of renal biopsies, and provide a reference for clinical treatment.

## Figures and Tables

**Table 1:** Basic characteristics of patients enrolled in all subcenters.

Variable	Total (*n* = 678)			Training group (*n* = 202)			Internal validation group (*n* = 87)			External validation group (*n* = 389)		
	DN (*n* = 295)	NDKD (*n* = 383)	P	DN (*n* = 103)	NDKD (*n* = 99)	P	DN (*n* = 47)	NDKD (*n* = 40)	P	DN (*n* = 145)	NDKD (*n* = 244)	*P*
Demographics												
Age (years)	53.3 ± 11.5	54.7 ± 12.0	0.124	53.2 ± 11.6	55.8 ± 12.4	0.125	53.8 ± 13.6	54.8 ± 11.9	0.392	53.0 ± 11.3	54.5 ± 11.6	0.212
Male (cases) (%)	225 (76.3)	242 (63.2)	<0.001	85 (82.5)	56 (56.6)	<0.001	37 (78.6)	26 (65.0)	0.229	103 (71.0)	160 (65.6)	0.313
BMI (kg/m^2^)	25.6 (23.8,28.3)	26.7 (23.8,29.2)	0.195	25.7 (23.9,28.1)	26.5 (23.4,29.2)	0.454	25.3 (23.7,29.5)	27.4 (24.7,29.3)	0.268	25.3 (23.3, 28.7)	27.2 (24.7, 29.7)	0.002
Medical history												
Diabetes onset age (years)	42.7 ± 11.2	49.5 ± 11.2	<0.001	43.0 ± 11.9	49.7 ± 10.3	<0.001	42.3 ± 9.9	49.3 ± 13.4	0.006	42.7 ± 11.1	49.4 ± 11.2	<0.001
Diabetes duration (years)	10.0 (5.0,15.2)	2.0 (1.0,8.5)	<0.001	10.0 (5.0,15.0)	2.0 (0.5,10.0)	<0.001	10.0 (8.0,20.0)	2.5 (0.5,7.8)	<0.001	10.0 (4.0,15.8)	2.0 (1,8)	<0.001
Hypertension history (cases) (%)	253 (86.1)	289 (75.5)	0. 001	88 (85.4)	72 (72.7)	0. 026	39 (83.0)	32 (80.0)	0. 786	126 (87.5)	185 (75.8)	0.006
Hyperlipidemia (cases) (%)	165 (56.1)	231 (60.3)	0.306	68 (66.0)	73 (73.7)	0.232	28 (59.6)	30 (75.0)	0.172	69 (47.9)	128 (52.5)	0.402
History of CHD (cases) (%)	53 (18.0)	58 (15.1)	0.346	17 (16.5)	15 (15.2)	0.792	12 (25.5)	5 (12.5)	0.176	24 (16.7)	38 (15.6)	0.776
Cerebrovascular history (cases) (%)	54 (18.4)	45 (11.7)	0.021	17 (16.5)	8 (8.1)	0.069	10 (21.3)	5 (12.5)	0.395	27 (18.8)	32 (13.1)	0.145
Smoking history (cases) (%)	160 (54.6)	151 (39.5)	<0.001	56 (54.9)	35 (35.4)	0.005	28 (59.6)	12 (30.0)	0.009	76 (52.8)	104 (42.8)	0.059
Drinking history (cases) (%)	109 (37.2)	128 (33.4)	0.329	42 (41.2)	27 (27.3)	0.038	12 (25.5)	12 (30.0)	0.038	55 (38.2)	89 (36.5)	0.745
Family history of T2D (cases) (%)	120 (40.8)	106 (27.8)	0.001	41 (39.8)	28 (28.3)	0.084	18 (38.3)	15 (37.5)	1.000	61 (42.4)	63 (26.0)	0.001
DR (cases) (%)	177 (60.2)	33 (8.6)	<0.001	57 (55.3)	6 (6.1)	<0.001	25 (53.2)	2 (5.0)	<0.001	95 (66.0)	25 (10.2)	<0.001
DPN (cases) (%)	54 (18.3)	16 (4.2)	<0.001	16 (15.5)	3 (3.0)	0.002	13 (27.7)	0 (0.0)	<0.001	25 (17.2)	13 (5.3)	<0.001
DMA (cases) (%)	116 (39.3)	92 (24.1)	<0.001	39 (37.9)	21 (21.2)	0.010	19 (40.4)	10 (25.0)	0.172	58 (40.0)	61 (25.2)	0.003
Laboratory result												
HbA1c (%)	7.0 (6.2, 8.1)	6.6 (6.2, 7.2)	0.001	6.7 (6.0, 7.8)	6.7 (6.3, 7.3)	0.865	6.9 (6.2, 7.9)	6.4 (6.0, 7.1)	0.082	7.4 (6.3, 8.2)	6.6 (6.2, 7.2)	<0.001
BUN (mmol/L)	10.1 (7.1, 14.2)	6.3 (5.0, 8.8)	<0.001	12.2 (7.8, 16.8)	7.3 (5.3, 9.5)	<0.001	11.2 (7.7, 15.8)	6.8 (5.1, 9.1)	<0.001	9.2 (6.6, 12.0)	6.0 (4.8, 8.3)	<0.001
Uric acid (*μ*mol/L)	406 (345, 474)	401 (335, 483)	0.240	412 (343, 479)	373 (304, 458)	0.020	398 (345, 462)	376 (340, 455)	0.562	407 (346.476)	408 (344.492)	0.852
eGFR (ml/min/1.73 m^2^)	43.5 (24.2, 67.1)	77.4 (49.5, 99.4)	<0.001	35.2 (18.8, 61.0)	64.4 (40.5, 94.3)	<0.001	40.2 (22.0, 62.9)	80.0 (48.4, 98.8)	<0.001	50.7 (32.1, 72.8)	79.9 (53.5, 101.6)	<0.001
Proteinuria (cases) (%)	142 (94.7)	129 (92.8)	0.628	100 (97.1)	90 (90.9)	0.063	42 (89.4)	39 (97.5)	0.212	140 (97.2)	228 (93.8)	0.152
Hematuria (cases) (%)	192 (65.3)	287 (74.9)	0.008	60 (58.3)	77 (77.8)	0.004	27 (57.4)	29 (72.5)	0.180	105 (72.9)	181 (74.2)	0.812
24 h UTP (g/24 h)	4.64 (2.42, 8.42)	3.50 (1.31, 7.24)	<0.001	5.04 (2.66, 8.84)	2.73 (0.94, 7.55)	0.008	4.08 (1.36, 9.28)	1.88 (0.55, 4.23)	0.011	4.65 (2.68, 8.09)	4.02 (1.47, 7.60)	0.037

Data are presented as percentages for categorical data, averages (*x* ± *s*) for continuous, normally distributed data, or medians (interquartile range) for continuous, nonnormally distributed data. Diabetes onset age refers to age at enrollment minus the duration of diabetes. When diabetes was newly diagnosed, the duration was recorded as 0 years, and the onset age was equal to the age at enrollment. The results of urine occult blood and urine glucose were stratified in routine urine examination. The test results of “+” – “+ + + +” are defined as hematuria and urine glucose positive. CHD: coronary heart disease; DR: diabetic retinopathy; DPN: diabetic peripheral neuropathy; DMA: diabetic macroangiopathy; BUN: blood urea nitrogen; eGFR: estimated glomerular filtration rate; 24 h UTP: 24-hour urinary total protein quantification.

**Table 2:** Univariate logistic regression analysis and multivariate logistic regression analysis of DN in the training group.

Risk factors	Univariate logistic regression analysis			Multivariate logistic regression analysis			
	OR	95% CI	*P*	*β* value	OR	95% CI	*P*
Diabetic retinopathy	19.207	7.713-47.829	<0.001	1.976	7.217	2.505-20.795	<0.001
Diabetes duration ≥ 5 years	5.662	3.027-10.590	<0.001	1.340	3.820	1.621-9.002	0.002
eGFR < 30 ml/min/1.73 m^2^	5.096	2.567-10.114	<0.001	1.076	2.932	1.033-8.324	0.043
High urea nitrogen	3.837	2.032-7.245	<0.001	0.030	1.031	0.394-2.699	0.950
Sex (male)	3.626	1.902-6.914	<0.001	1.076	2.932	0.922-9.324	0.068
Diabetes onset age ≤ 40 years	3.147	1.683-5.888	<0.001	0.521	1.683	0.688-4.118	0.254
24 h UTP ≥ 3 g	2.535	1.418-4.532	0.002	0.965	2.626	1.064-6.482	0.036
No hematuria	2.508	1.357-4.638	0.003	1.161	3.192	1.258-8.100	0.015
Hyperuricemia	2.252	1.246-4.069	0.007	0.659	1.932	0.800-4.666	0.143
Smoking history	2.226	1.262-3.926	0.006	0.188	1.207	0.464-3.141	0.700
History of hypertension	2.200	1.088-4.447	0.028	0.115	1.121	0.429-2.935	0.815

High urea nitrogen: >7.1 mmol/l; hyperuricemia: uric acid >360 *μ*mol/L; diabetes onset age refers to age at enrollment minus the duration of diabetes; when diabetes was newly diagnosed, the duration was recorded as 0 years, and the onset age was equal to the age at enrollment. The results of urine occult blood were stratified in routine urine examination, and the test results of “+”– “+ + + +” were defined as hematuria.

**Table 3:** Multivariate logistic regression analysis of risk factors for DN in the training group and diabetic nephropathy risk score model.

Risk factors	*β* value	OR	95% CI	*P*	Value
Diabetic retinopathy	2.188	8.913	3.343-23.766	<0.001	2
Diabetes duration ≥ 5 years	1.360	3.896	1.804-8.411	0.001	1
eGFR < 30 ml/min/1.73 m^2^	1.014	2.758	1.166-6.524	0.021	1
24 h UTP ≥ 3 g	1.218	3.382	1.527-7.491	0.003	1
Hematuria (none)	1.176	3.242	1.376-7.640	0.007	1

**Table 4:** Proportion of DN patients in different risk groups.

Risk level	Score	Proportion of DN patients		
		Training group	Internal validation group	External validation group
Low-risk	0-1	15.1% (11/73)	6.5% (2/31)	8.5% (13/153)
Medium-risk	2-3	54.2% (38/70)	64.5% (20/31)	41.6% (57/137)
High-risk	4-6	91.5% (54/59)	100% (25/25)	81.9% (68/83)

## Data Availability

Due to the limitations of the “Regulations on the Management of Human Genetic Resources of the People's Republic of China,” this study is unable to provide open raw data.
